# A mixture framework for inferring ancestral gene orders

**DOI:** 10.1186/1471-2164-13-S1-S7

**Published:** 2012-01-17

**Authors:** Yiwei Zhang, Fei Hu, Jijun Tang

**Affiliations:** 1 Center for Computational Biology, Beijing Forestry University, Beijing 100083, China; 2 Department of Computer Science and Engineering, University of South Carolina, Columbia, SC 29208, USA

## Abstract

**Background:**

Inferring gene orders of ancestral genomes has the potential to provide detailed information about the recent evolution of species descended from them. Current popular tools to infer ancestral genome data (such as GRAPPA and MGR) are all parsimony-based direct optimization methods with the aim to minimize the number of evolutionary events. Recently a new method based on the approach of maximum likelihood is proposed. The current implementation of these direct optimization methods are all based on solving the median problems and achieve more accurate results than the maximum likelihood method. However, both GRAPPA and MGR are extremely time consuming under high rearrangement rates. The maximum likelihood method, on the contrary, runs much faster with less accurate results.

**Results:**

We propose a mixture method to optimize the inference of ancestral gene orders. This method first uses the maximum likelihood approach to identify gene adjacencies that are likely to be present in the ancestral genomes, which are then fixed in the branch-and-bound search of median calculations. This hybrid approach not only greatly speeds up the direct optimization methods, but also retains high accuracy even when the genomes are evolutionary very distant.

**Conclusions:**

Our mixture method produces more accurate ancestral genomes compared with the maximum likelihood method while the computation time is far less than that of the parsimony-based direct optimization methods. It can effectively deal with genome data of relatively high rearrangement rates which is hard for the direct optimization methods to solve in a reasonable amount of time, thus extends the range of data that can be analyzed by the existing methods.

## Background

Inferring gene orders and gene content of ancestral genomes has a wide range of applications. High-level rearrangement events such as inversions, transpositions that change gene order are important because they are "rare genomic events" [1] and can be used to estimate ancestral genomes and infer the number of steps that evolve one genome into another.

The fundamental question in building phylogenies is how far apart two species are from each other. Hannenhalli and Penvzner [2] provided the first polynomial algorithm for computing inversion distance, which can be used as a representation of the evolution distance between species. Then by using a direct optimization approach based on median calculation, which is to optimize each ancestral node in terms of its three or more immediate neighbors, the phylogeny and ancestral genomes could be reconstructed. Current popular methods (such as GRAPPA [3], MGR [4] and their new improved versions) all use this approach and can infer ancestral gene orders with high accuracy. However these methods are extremely slow especially when the rearrangement rate is high. A new method based on the maximum likelihood approach is recently proposed [5]. In this method, the probabilities of all possible ancestral gene order are calculated based on the present species' gene order data and the ancestral gene orders are reconstructed to maximize the overall probability. This method is much faster than the direct optimization methods under high evolution rearrangement event rate, but the accuracy of the reconstructed gene order is lower than that of the direct optimization methods.

In this paper, we propose a mixture method to enhance the inference of ancestral gene orders. We first use the maximum likelihood method to reconstruct an initial ancestral genome. Then we randomly select a number of gene adjacencies from the ancestral genome and fix them to run the median calculation. By analyzing the results of each median calculation, we try to infer the correct gene adjacencies generated by the maximum likelihood method. Finally we fix these adjacencies and perform median calculation to get the ancestral genome. We have conducted extensive experiments on simulated datasets and the results show that this mixture approach is more accurate than the maximum likelihood method. Also our method is much faster than solely using the median calculation when the rearrangement rate is high. Using this hybrid approach, for those datasets that are previously too difficult for existing methods, we will be able to analyze them within a reasonable time frame with very high accuracy.

### Genome rearrangements

Given a set *S *of *n *genes {1, 2, ⋯, *n*}, a genome can be represented by an ordering of these genes. A gene with a positive orientation is written as *i*, otherwise it is written as a *-i. *A genome can be *linear *or *circular*. A linear genome is a permutation on the gene set, while a circular genome can be represented in the same way under the implicit assumption that the permutation closes back on itself. Let *G *be the genome with signed ordering of 1, 2, ⋯, *n*. An *inversion *(also called reversal) between indices *i *and *j *(*i *≤ *j*), transforms *G *to a new genome with ordering

1,2,⋯,i-1,-j,-(j-1),⋯,-i,j+1,⋯,n

There are some additional events for multiple-chromosome genomes, such as *translocation *(when the end of one chromosome switches with the end of another chromosome), *fission *(when one chromosome splits to form two) and *fusion *(when two chromosomes combine to become one).

**Figure 1:**
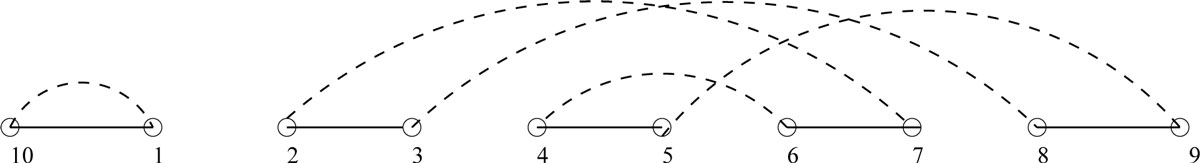
**An example of breakpoint graph.** Breakpoint graph of genome *G *= 1,2,-3,4,-5, with respect to the identity genome *G *= 1,2,3,4,5.

We use a breakpoint graph [6] to represent the permutation with respect to the identity permutation. Given a genome with permutation *π*, let the breakpoint graph that corresponds to it be *M *(*π*). The vertex set *V *of *M *(π) is the collection of {*2i *- 1, *2i*}, and *i *is any distinct gene of permutation *π*. Two genes *i *and *j *are said to be adjacent in genome *G *if *i *is immediately followed by *j *and can be represented by an edge (2*i*, 2*j*-1). If *i *and -*j *are adjacent, then it can be represented by an edge (2*i*, 2*j*). The edge set *E *of *M *(*π*) consists of all the adjacencies in *π*. For example, for a circular unichromosomal genome *G*_2 _= 1,4, 2, -3, 5, the vertex set is *V *= {1, 2, 3, 4, 5, 6, 7, 8, 9,10} and the edge set is *E *= {(10,1), (2, 7), (3, 8), (4, 6), (5, 9)}, as shown in Figure 1. The breakpoint graph extends naturally to a multiple breakpoint graph (MBG), representing a set of three or more genomes.

### Inferring ancestral gene order by median calculation

One popular approach to reconstruct ancestral genome is through direct optimization and the task is to seek the tree with the minimum number of events, which is in spirit similar to the maximum parsimony approach used in sequence phylogeny. The core of these methods is to solve the median problem defined as follows: given a set of *N *genomes with permutations {*π*_*i*_}_1≤*i *≤ *N *_and a distance measure *d*, find a permutation *π*_*M *_that minimizes median score defined as $\sum_{i=1}^{N}d\left(\pi_{i},\pi_{M}\right)$.

Widely used methods like GRAPPA [3] and MGR [7] (as well as their new improved versions) both use median calculation to infer phylogenies and ancestral gene orders. For example, GRAPPA examines every possible tree topology and reports the one with the minimum events. For a given tree, it iteratively updates the gene order on each ancestral node in terms of its three neighbors through median calculation until the whole score of the tree reaches minimum. At this point the gene order obtained on each internal node corresponds to the inferred ancestral genome data and the total number of events is simply the summation of pairwise distances along all edges.

The median problem is known to be NP-hard [6] under most rearrangement distances proposed so far. Recently Xu proposed the concept of Adequate Subgraph [8] and applied this theory to create the ASMedian solver, which is the fastest median solver to date. The Multiple Breakpoint Graph (MBG) is used to model the median problem and an Adequate Subgraph is defined as a subgraph that has number of cycles larger or equal to 3*m*/2 (*m *is half the number of the vertices of the subgraph). Adequate subgraphs allow a decomposition of an MBG into smaller, more easily solved graphs. The median solution is the combination of the median solutions for the adequate subgraphs and the remaining MBG, so we can shrink the known median edges of the adequate subgraph and reduces the original MBG size.

**Figure 2:**
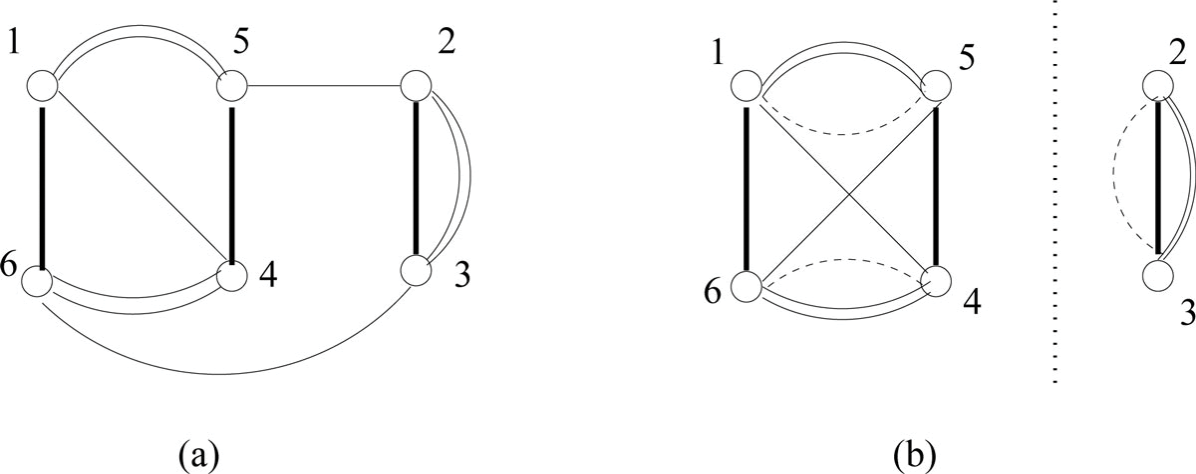
**An example of adequate subgraph. **(a) An MBG based on three circular genomes {1, 2, 3} (thick lines), {1, 2, -3} (double lines), {1, 3, 2} (thin lines). (b) The reduced MBG with (2 3) shrunk because (2, 3) is an adequate sub-graph. The dash line indicates the median solution 1, 2, 3, which is the combination of the median solution of the two sub-graphs.

Figure 2 shows an example of adequate subgraph decomposition. Given three circular genomes {1, 2, 3}, {1, 2, -3} and {1, 3, 2}, the MBG of these three genomes are shown on the left. Vertices (2, 3) composes an adequate subgraph according to the definition of adequate subgraph, so we can shrink (2, 3) from the MBG and get a reduced MBG as shown on the right. The final median solution is also shown in the figure and we can see that the edges of the median solution are within the reduced MBG.

By searching the existing adequate subgraphs up to a certain size and incorporate them into an exhaustive algorithm for the median problem, the ASMedian solver could significantly reduce the computation time of median calculation, though it still runs very slowly with high gene rearrangement rate. Given *n *the number of genes in the dataset and *r *the expected number of events per edge, when the ratio of *r*/*n *is larger than 25%, all direct optimization methods have great difficulty in finishing the analysis even after months of computation. As ASMedian (as well as other median algorithms) use a branch-and-bound approach, its performance relies on how to quickly prune branches. If we can fix some adjacencies before performing median calculation, the search space will be further decreased and the problem would be solved much faster.

### Reconstruct the ancestral genomes based on maximum likelihood

Another new approach to reconstruct the ancestral genome is based on maximum likelihood. Ma proposed a probabilistic framework for inferring ancestral genomic orders with rearrangements including inversion, translocation and fusion [5]. When the phylogeny tree is given, the tree is re-rooted so that the ancestral node to be inferred becomes the root of the new tree. In this way, all the leave nodes can be taken into account for reconstructing the target ancestral node.

Because the gene order of any ancestral node is unknown, the probabilities of all possible gene adjacencies within the genome need to be calculated. Since each internal node of the phylogeny tree has two children and we assume that both children evolved from the parent node, the posterior probability of any gene adjacency within the parent node can be computed when treating the gene orders of the children as observed data. The calculation can be performed recursively from the bottom leaves level to the top level until the root is reached. At this point the probabilities of all the possible gene adjacencies within the target ancestral genome are computed. Finally an approximate algorithm is used to pick up and connect the adjacencies together to maximize the overall probability, and these selected adjacencies form the final inferred ancestral genome.

## Methods

We propose a framework that combines both maximum likelihood and direct optimization approaches to infer ancestral gene orders. The maximum likelihood method runs much faster with high genome rearrangement rate compared with the direct optimization methods, but the latter can infer more accurate gene orders. To speed up the latter, we need to reduce the computation time for the median calculation as it is clearly the bottleneck of the computation. Our method tries to pick up the correctly inferred gene order from the maximum likelihood method and fix these adjacencies in the median calculation. As a result, the median search space can be significantly reduced and the median calculation is accomplished much faster while the accuracy of the inferred gene order is also improved.

### Reduce median search space by fixing adjacencies

**Figure 3:**
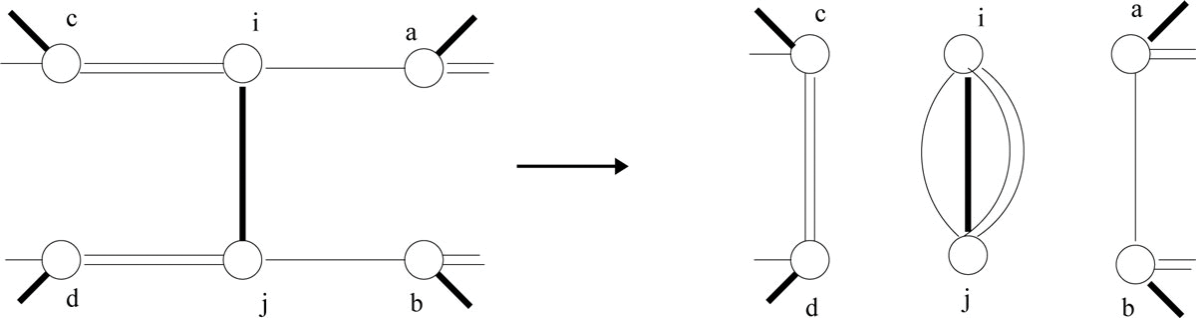
**Fix an adjacency (*i*,*j*) in the MBG.** On the left is the MBG with three permutations represented by thin lines, double lines and thick lines respectively. On the right is the MBG with (*i*,*j*) fixed for all the permutations.

We use Xu's ASMedian solver which is based on the Adequate Subgraph theory [8]. In order to speed up the median calculation, we have to further reduce the median search space. Given permutation {*π*_*i*_}_1≤*i*≤*N*_, let the MBG that corresponds to it be *M *(*π*). If we assume an adjacency (*i*, *j*) should be present in the final median solution, we could force {*i*, *j*} to become an adequate subgraph of *M *(*π*). The procedure is as follows: For each permutation *π*, if edge (*i*, *j*) is already present in the breakpoint graph, we do nothing. Otherwise let (*i*, *a*), (*j*, *b*) be the edges corresponding to vertices *i *and *j*. We then remove both edges (*i*, *a*), (*j*, *b*) and create two new edges (*a*, *b*), (*i*, *j*). Figure 3 shows the procedure stated above. Three permutations are represented by thin lines, double lines and thick lines respectively. For the permutation of thick lines, edge (*i*, *j*) already exists, so nothing needs to be done. For the other two permutations, edge (*i*, *j*) is not present. For the permutation represented by double line, we remove the original edges (*c*, *i*) and (*d*, *j*) which are incident to vertices *i*, *j *and add an edge (*i*, *j*) with double line. For the permutation represented by thin line, we remove the original edges (*a*, *i*) and (*b*, *j*) which are incident to vertices *i*, *j *and add an edge (*i*, *j*) with thin line. According to the definition of adequate subgraph, vertices {*i*, *j*} become an adequate subgraph because three edges are incident to these two vertices [8].

If we can fix a number of adjacencies, an equal number of adequate subgraphs can be created. Thus we can shrink these subgraphs from the original multiple break point graph, and the original median problem will be greatly reduced. If the adjacencies that we try to fix are the actual adjacencies present in the final solution, this fixing procedure will not affect the accuracy of the median solution.

### Infer the correct adjacencies

Based on our observation and simulation, only a part of the adjacencies inferred by the maximum likelihood method are the correct adjacencies that are present in the ancestral genome. It will be desired if we can optimally pick out all the correct adjacencies and fix them for the median computation. However, it is very difficult to find out all the correct adjacencies inferred by the current maximum likelihood method as only a small percentage of adjacencies are reported with high probability while others usually have similar small probability which cannot be used to judge whether an adjacency is correct or not. Our method to identify the correct adjacencies is a randomized approach. First, we will keep adjacencies that have higher probabilities as "correct". Then we will randomly select a number of adjacencies from those with lower probability and fix them in the median computation using ASMedian. These randomly selected adjacencies may contain both correct and incorrect adjacencies, but the correct adjacencies are more likely to lead the inference of other correct adjacencies within the ancestral genome after the median computation. Thus after the median is found, we examine the non-fixed adjacencies in the "ancestral genome" generated by the median solver and record the ones that also appear in the "ancestral genome" generated by the maximum likelihood method. We repeat the procedure many times. Finally for each adjacency generated by the maximum likelihood method, we get the number of times it appears in the resulted median genomes. The more it appears, the more likely it is a correct adjacency.

The number of randomly selected adjacencies should not be too large. Too many fixed adjacencies may contain too many incorrect adjacencies to pollute the median calculation and leads to bad results. On the other hand, the number of fixed adjacencies should not be too small, otherwise the time consumption of median calculation is unacceptable since the search space is still too large. In our practice, we randomly choose 70-75% of the adjacencies and fix them in the median computations. We also conducted extensive testing to verify that 70-75% is indeed a good choice and the results are shown later.

When more than three genomes are involved and a phylogeny is given, we will first infer part of the correct adjacencies for each ancestral genome that corresponds to an internal node. We will then use our modified median solver to iteratively update the gene order of these internal nodes with those fixed adjacencies. The tree edge length is the genomic distance between the nodes and the score of the tree is defined as the sum of all the tree edge. The iteration stops when the score of the tree dose not change, suggesting that convergence is reached. At this time, gene orders contained in the internal nodes are the final ancestral genomes to be reported.

## Results and discussion

### Experimental methods

We use simulated data to assess the quality of our method as we know the "true" ancestors in simulations. In our experiments, we use two models to generate the data. First we generate model tree topologies from the uniformly distributed binary trees, each with 10 leaves indicating 10 genomes. We use different evolutionary rates, of 20%, 24% ... 36% expected rearrangement rates per tree edge, with 50% relative flucturation. We also use the model proposed by Lin and Moret [9] to generate another set of random trees with 10 leaves. The tree diameters range from 2.0 to 4.0. The Lin and Moret's tree model has more unbalanced edge lengths and is suggested as more biologically realistic.

Once the simulated genomes are generated, we use our mixture method to infer the ancestral gene order data. Because we are handling genomes with equal gene contents, the accuracy can be measured with the number of correctly inferred adjacencies (the inferred adjacencies that also appear in the original ancestral genome) divided by the total number of adjacencies for each genome.

### Number of replicates required

As discussed before, we use maximum likelihood method to reconstruct the initial ancestral genome and then randomly select a number of gene adjacencies from the initial ancestral gene order data and fix them for median computation. This procedure is repeated until each adjacency has been selected for enough times. Finally we record the number of occurrences of each gene adjacency that are present in the median results (excluding the case when it is fixed in median computation) and sort these adjacencies according to the number of occurrences. If the number of repeats is large enough, the sorting of the gene adjacencies will remain almost unchanged.

According to our test, when the number of replicates is over 100, the sorting of the gene adjacencies comes to a steady state and there is little change with more repeated tests are conducted. Since we will pick a certain percentage of adjacencies with higher numbers of occurrences, the very small change in adjacency sorting will hardly affect the result.

### Adjacency selection percentage

When we sort the gene adjacencies by the number of their occurrences in the median calculation result, it is clear that the more an adjacency appears, the more it is likely to be a correct adjacency. Thus we can just pick the adjacencies with large numbers of occurrences and fix them in the median calculation. However, we need to determine the threshold of how many adjacencies should be fixed. We define the *selection percentage *as the number of selected adjacencies divided by the total number of adjacencies. From our experience, we found that the percentage cannot be lower than 65%, otherwise the median calculation are still very slow as the search space is not small enough.

To determine the selection percentage, we use various percentages, from 65% to 85%, to select the gene adjacencies and fix them to reconstruct ancestral genomes through median computation. We compare the average accuracy of the reconstructed gene order under different selection percentages. Table 1 shows the average accuracy of reconstructed gene order under different selection percentages, using uniform trees with various evolutionary rates per edge, while Table 2 shows the results of Lin and Moret's tree model with various tree diameters.

**Table 1:** Accuracy of reconstructed gene order (%) under various selection percentages and rearrangement rates with uniformly distributed tree model

	65% selected	70% selected	75% selected	80% selected	85% selected
20% events per edge	89.73	91.93	90.47	89.53	89.24
24% events per edge	80.28	81.92	81.07	80.56	79.81
28% events per edge	71.59	73.12	73.28	71.10	71.63
32% events per edge	63.33	64.52	65.72	65.25	64.81
36% events per edge	59.45	60.87	62.43	60.27	60.15

**Table 2:** Accuracy of reconstructed gene order (%) under various selection percentages and diameters with Lin's random tree model

	65% selected	70% selected	75% selected	80% selected	85% selected
Diameter = 2.0	93.49	94.37	93.97	93.01	92.89
Diameter = 2.5	89.02	89.93	89.02	88.31	88.18
Diameter = 3.0	84.75	86.51	85.25	84.58	84.45
Diameter = 3.5	80.21	82.04	82.93	80.58	80.50
Diameter = 4.0	79.14	79.84	80.96	79.37	78.75

From these tables, we can see that when the rearrangement rate or the tree diameter is relatively small (meaning genomes are closely related), the gene accuracy reaches maximum values when the selection percentage is 70%. When the rearrangment rate or the tree diameter grows larger, it would be better to use 75% as the selection percentage. This is because with larger rearrangement rates, some correct adjacencies inferred by the maximum likelihood method are hard to be inferred by the median calculation, so we should use a larger selection percentage to include more adjacencies inferred by the maximum likelihood method.

### Accuracy on closely related genomes

First we compare the accuracy of the ancestral genome reconstructed by our method with the result from both Ma's maximum likelihood approach and the pure ASMedian solver. Because ASMedian solver is very time consuming, we only perform tests under lower rearrangement rates and diameters. We perform simulation tests using the two types of tree models as stated above with various rearrangement rates and tree diameters. For each configuration we run 50 data sets and the results are averaged. Table 3 shows the comparison under uniform distribution binary trees with average rearrangement rates ranging form 12% to 20% and Table 4 shows the comparison under Lin's random trees with diameters ranging form 0.6 to 1.2. As mentioned before, we can see that the median method produces more accurate results than those of the maximum likelihood method under all situations. Also we notice that our mixture method could produce a similar or even slightly better result than solely using ASMedian. These tables also show that the randomized approach could effectively infer correct adjacencies from the maximum likelihood method and leads to equivalent accuracy of the best median solver.

**Table 3:** Reconstructed gene order accuracy comparison with low rearrangement rates under uniformly distributed tree model

	Ma's method	Pure median method	Our mixture method
12% events per edge	92.28	98.53	98.49
16% events per edge	86.37	96.25	96.12
20% events per edge	81.54	91.76	92.13

**Table 4:** Reconstructed gene order accuracy comparison with small diameters under Lin's random tree model

	Ma's method	Pure median method	Our mixture method
Diameter = 0.6	96.75	99.37	99.12
Diameter = 0.9	95.48	97.62	97.31
Diameter = 1.2	92.39	95.81	95.89

### Accuracy on distant genomes

ASMedian solver cannot finish datasets with higher rates or diameters, thus for distant genomes, we only compare the accuracy of the ancestral genome reconstructed by our method with the result of Ma's maximum likelihood approach. To see the effectiveness of our new approach, we also compare the optimal solution when all correct adjacencies in the maximum likelihood results can be told, which is impossible in real data analysis but is trivial in simulation as the truth is known. This optimal solution is also the upper bound that our mixture framework can achieve.

**Figure 4:**
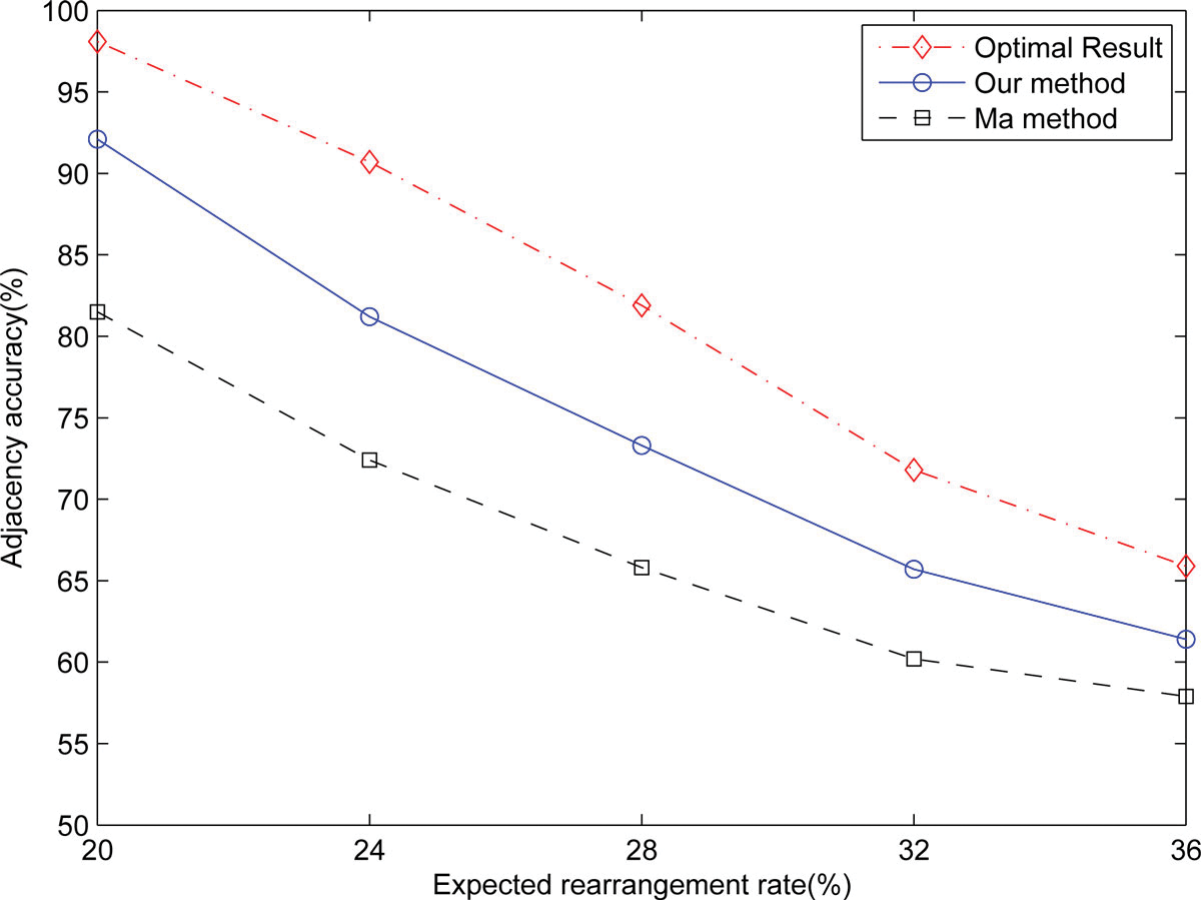
Comparison of the reconstructed gene adjacency accuracy with high rearrangement rates under uniform distribution binary trees.

Figure 4 shows the comparison under uniform distribution binary trees with different rearrangement rates per edge. We can see that our method can infer ancestral gene order more accurately than Ma's method. At the rearrangement rate of 20% we get an average improvement of nearly 12%. With the increase of rearrangment rates, the improvement decreases, but we still have about 4% in adjacency accuracy comparison when the rearrangement rate reaches 36%. Compared with the optimal median result, the accuracy of our method are about 10% less at the rearrangement rate of 20%. The difference decreases when the arrangement increases, and we can see that the result of our method is still close to the optimal result.

**Figure 5:**
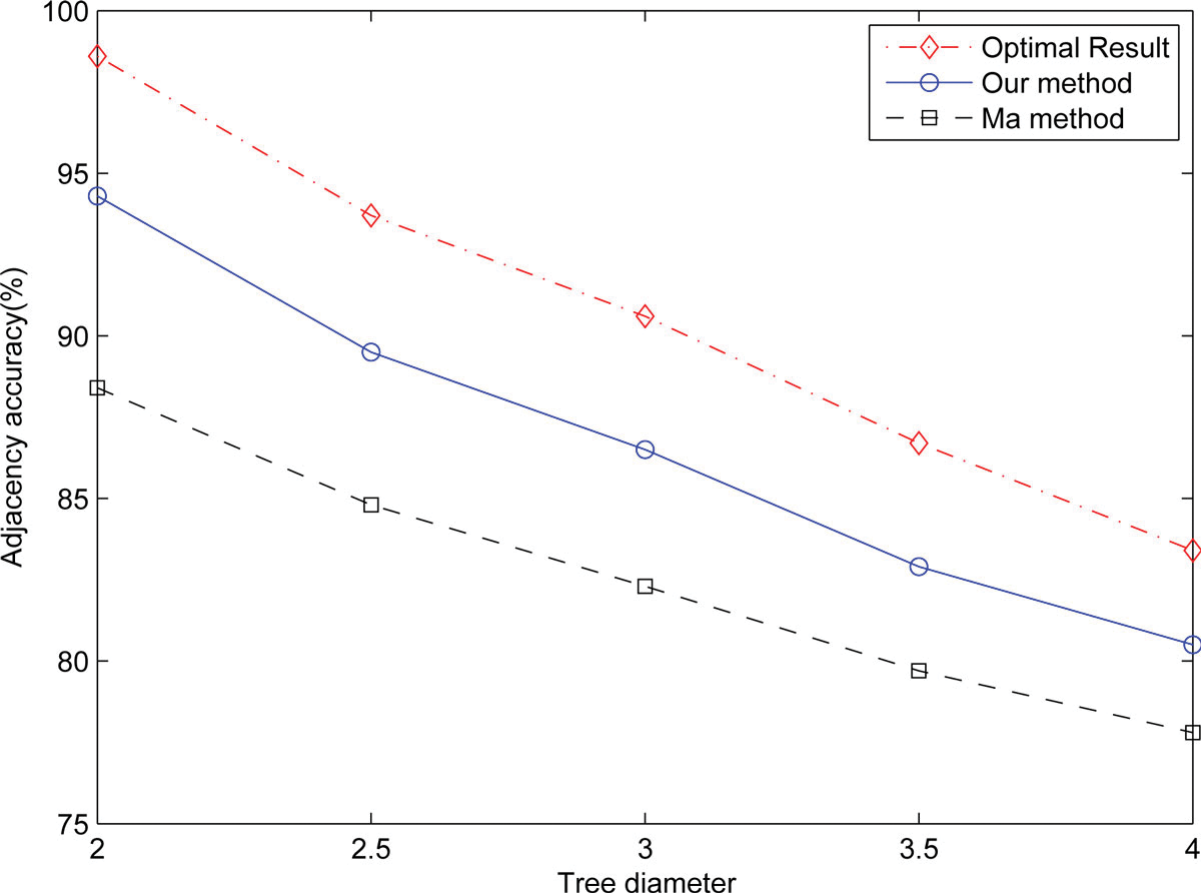
Comparison of the reconstructed gene adjacency accuracy with high rearrangement rates under Lin's random tree model.

Figure 5 shows the comparison under random trees with different diameters. Because of more unbalanced edge lengths which decrease the accuracy of the gene order inferred by the median calculation, the improvement of the adjacency accuracy is not as much as the uniform tree model. But still we have an average improvement of about 7% with diameter equals 2.0. With the increase of diameter, the improvement decreases and finally we still have more than 3% when diameter equals 4.0. Compared with the optimal result, the accuracy of our method are about 5% less when the diameter is 2.0. The difference decreases when the diameter increases, and we can see that under random tree models with very unbalanced edge lengths, the difference between our result and the optimal result are much smaller than that of the uniform distributed tree model.

In conclusion, our method that randomly picks adjacencies from the maximum likelihood method could achieve accuracy that is comparable to the optimal result by picking all the correct adjacencies. For distant genomes, the original median-based parsimony methods cannot give result in reasonable time and we can expect that the result of parsimony method will not exceed the optimal result achieved by fixing part of the correct adjacencies. As a result, we assume that our method could achieve similar accuracy for distant genomes compared with the parsimony methods. Also the result shows that, although the accuracy decreases when genomes are more distant from each other, our method are still more accurate than the original maximum likelihood method. However, for very distant genomes (rearrangement rate over 40% or diameter over 4.5), the maximum likelihood method can only produce result with very low accuracy, resulting not enough correct adjacencies for our mixture method. If we keep the original selection percentage (70% to 75%), the accuracy of the inferred ancestral genome becomes very low. On the other hand, if we use lower selection percentages, it will be very difficult for the median calculation to finish in reasonable time as not so many adjacencies are fixed. Thus inferring the ancestral genome for very distant genomes is still a challenging problem for our method.

### Time consumption

We also compare the time consumption of our mixture method and the original parsimony method. The time cost of our mixture method includes the time spent on the maximum likelihood method and the time for median calculations with multiple fixed adjacencies. As stated above, operations for calculating median with randomly selected adjacencies are independent from each other, so they can be performed in parallel. The test is performed on an server with 2.4GHz, 4 core CPUs. Average running time of the two methods is reported in seconds.

Table 5 shows the comparison under uniform distribution binary trees with different rearrangement rates per edge. Table 6 shows the comparison under random trees with different diameters. The speedups range from 1.8 to over 139 or even more, increasing along as the rearrangement rates or tree diameters increase. Also we can see that for distant genomes with rearrangement rate over 24% or diameter over 2.5, the original parsimony method based on ASMedian solver cannot finish the computation within reasonable time while our method can still accomplish computation in relatively short time. As shown in this table, our mixture method achieves significant speedups compared with the original parsimony method.

**Table 5:** Comparison of average time cost under uniform distribution binary trees

Rearrangement rate per edge	16%	20%	24%	28%	32%
Pure median method	315	15800	1.4 × 10^5^	N/A	N/A
Our mixture method	180	760	1270	1620	1850
Speed up	1.75	20.78	110.23	N/A	N/A

**Table 6:** Comparison of average time cost under Lin's random tree model

Tree diameter	1.5	2.0	2.5	3.0	3.5
Pure median method	8500	76400	2 × 10^5^	N/A	N/A
Our mixture method	430	840	1430	1920	2230
Speed up	19.76	90.95	139.86	N/A	N/A

## Conclusions

Ancestral gene order data can be inferred by either median computations or the maximum likelihood method. Median calculation can give a more accurate result but is extremely time consuming under high rearrangement rates. Maximum likelihood method runs much faster but the inferred gene order is not as accurate as the result of median method. We propose a mixture method to infer the ancestral gene order based on the above two approaches to improve both time cost and accuracy. The main idea of our method is to pick the correct gene adjacencies through a randomized procedure and reduce the median computation cost by fixing these correct gene adjacencies. Experiments show that our mixture method produces more accurate ancestral genomes than the maximum likelihood method while the computation time is far less than that of pure median method.

## Competing interests

The authors declare that they have no competing interests.

## Authors' contributions

YZ and JT contributed to the development and implementation of the algorithms, and FH were in charge of conducting simulations.
